# The patient as a policy problem: Ambiguous perceptions of a critical interface in healthcare

**DOI:** 10.1177/1363459320976757

**Published:** 2020-12-08

**Authors:** Peter Garpenby, Ann-Charlotte Nedlund

**Affiliations:** Linköping University, Sweden

**Keywords:** health policy making, patients, policy style, problem frames, Sweden

## Abstract

The interface between the patient and the health service has changed, which constitutes a potential problem for various policy-makers. Using a critical policy perspective and drawing on the theory of problem framing, this paper explores how actor groups with different responsibilities perceive the patient as a constructed policy problem. This is a qualitative study where data consists of single episode interviews with healthcare politicians, senior administrators, service strategists, and unit mangers from one regional health authority in Sweden. A thematic content analysis of the interviews was carried out in accordance with “the framework approach”. The study illustrates how the actors interpret their reality using diverse problem frames. This becomes more visible when the framing is disentangled with regard to what perspective they employ in relation to different accounts: society or the individual, or the (healthcare) system or the (healthcare) professional. The actor groups are part of the same institutional context, which explains certain tendencies of similarities in terms of the accounts being used, but still they approach the constructed problem differently which is visible as shifts—scaling up and down—between different accounts. By analyzing and structuring the various problem frames (including its policy styles) we can enhance our knowledge about how those responsible for the governance of healthcare approach the patient as a policy problem, as something that concerns only the patient and/or the provider, or as something that needs to be addressed in broader strategic terms.

## Introduction

Viewed from a longer-term perspective, patients per se have seldom been at the center of health policy. The foundation of publicly funded healthcare systems, like the British NHS and the regionalized health services in the Nordic countries, was the institutionalization of, in the words of Klein (2013), “technocratic paternalism.” When welfare state arrangements were expanded in post-war Europe, the focus was not on patients as recipients of care but rather on patients as citizens who were given access to care—a result of the public making its voice heard as voters, or organized in politics. It was this collective body which was granted extended rights of entitlement (Dent and Pahor, 2015; Freeman, 2000; Moran, 1995). Within a broad politically determined framework, the business of managing patients was left to the doctors and their authority—a relationship that remained virtually unregulated (Dent and Pahor, 2015).

Nowadays, the interface between health services and patients is more fluid, which presents a potential dilemma for policy-makers as well as practitioners. Rising service expectations from citizens and patients with regard to access, quality and involvement are increasingly set against limitations on financial resources and personnel (Saltman, 2015). If the patient starts to be regarded as an actor, or even as a co-producer of care, as opposed to a recipient of care, this will impact on the conditions for health policy-making (Vermeulen and Krabbe, 2018). How this is manifested depends on whether the change is understood as something that concerns only the meeting between the individual (patient) and the provider or if it is interpreted in broader strategic terms, as something important to the health system at large and its future design (Klein, 1993; Osborne and Strokosch, 2013; Salter, 2003; Vrangbaek, 2015). Policy-makers, whether they are elected or unelected officials, managers or front-line professionals, have to relate to new societal values, expectations, and behavior among patients.

In this paper, we look into the different problem frames among varied policy-makers in publicly funded healthcare. The setting of this study is a Swedish regional health authority where directly elected politicians, advised by senior administrators, decide on the allocation of services within broad national framework legislation. The politicians not only sit on the directly elected political assemblies that decide on the overall budgets, but also make up the standing committees for commissioning health services and deciding on major investments (Garpenby and Nedlund, 2016; Hagen and Vrangbæk, 2009). Within this frame, healthcare is to be organized and delivered by managers and professionals to patients with divergent health-related problems and preferences.

Still, the making of policy, as directed toward large groups, is by necessity the simplification of problem-solving. The process for constructing policy problems and categorizing groups of citizens is a prominent element in the political and the policy process (Nedlund and Nordh, 2018; Schneider and Ingram, 2005). In terms of health policy-making, patients—particularly in universal systems, although this is a highly heterogeneous group—must largely be treated as a fairly homogeneous mass with regard to entitlement and services. Over time, when individual rights and personalization are added to the political agenda, the balance between standardization of the system on the one hand and professional and patient leeway on the other increasingly creates dilemmas for the various policy-makers.

The policy-makers in healthcare are not unitary; their position and perspective differ, as do their responsibilities with regard to the making of policy. The conditions for making policy are different for elected officials (politicians), who are responsible for the overall population budget, compared to managers, who are responsible for a fixed budget, or professionals in clinical units. In a formal sense, the politicians have vast power resources, as they are drawing on a democratic mandate and thus are controlling the tax money, which underpins public healthcare. However, as elected representatives, they depend on public opinion and they lack important channels into the professional organization. The senior administrators are the links between politicians and important knowledge sources. As the administrators are in regular contact with the provider organization this link will become an important source of power. The unit managers have access to one important knowledge source, the patients, but are tied by decisions made elsewhere. They are, however, part of a strong professional organization. The service strategists lack formal power but have important insights into the healthcare organization—they observe the interplay between staff and patients. Hence, it can be assumed that their perceptions of the patient will differ, which in itself may have implications for the making and execution of health policy (Dent and Pahor, 2015; Harper and Honour, 2015; Vrangbaek, 2015).

The objective of this paper is to explore how four actor groups with different responsibilities as policy-makers perceive the patient as a constructed policy problem in a public healthcare system. Based on interviews with politicians, senior administrators, clinical unit managers, and service strategists at one Swedish regional health authority, this study offers insights into how the framing of a policy problem will differ among actor groups. By analysing and structuring the various policy frames including their policy styles, we can enhance our knowledge about how these framings represent shifts—scaling up or down—between different accounts.

## Policy problem, problem frames and policy styles

One way to understand the interface between the patient and the health service is to take a critical policy perspective. From such a perspective, the patient can be regarded as a policy problem where the process for constructing policy problems and categorizing groups of citizens into policy targets is a regular element in the policy process (Greenhalgh and Russell, 2009; Schneider and Ingram, 2005; Stone, 2002). A policy problem is not just “there”; it does not follow pure rationality, and it is often constructed before the goals are formulated, by the goals or after a solution has been identified. Both the means and the goals are subject to discussion, where people with social roles—and not abstractions of actors—interact and negotiate ideas and values (Nedlund and Nordh, 2018; Schneider and Ingram, 1997; Stone, 2002) in an attempt to come to shared understandings about how to handle collective concerns appropriately (Colebatch, 2006; Nedlund, 2012).

Rein and Schön, (1996), Schön and Rein (1994) understand the constructions of policy problems and preferred solutions to be drawn on different problem frames—underlying structures of belief, perception, and appreciation (Schön and Rein, 1994: 23).These frames are created in processes of naming features of a situation that highlight and select what should be seen (as a contrast to other features), and that are presented in a situational story. In this process, both the familiar and the unfamiliar come to be seen in new ways (Schön and Rein, 1994: 27). In a policy controversy, opposing actors have different stories and thus different views of reality. They hold conflicting frames, which convey which facts are “true” and which arguments are relevant. Hence problem frames, in terms of naming features of a situation, shape policy positions but also create what will be seen as a policy problem.

Problem-framing is about selecting and simplifying vague or complex problematic situations. It can be understood as a dynamic questioning process (Turnbull, 2013) related to how strong the questioning and answering is, as well as the legitimation of the actors’ own positions in their particular field (whether political, bureaucratic, managerial, professional, etc.). The patient as a policy problem in a healthcare setting is thus related to the different approaches the various social actors continuously take to the constructed problem.

In policies, following the taxonomy of Nedlund and Nordh (2018), various competing categories of rationales can be identified: a technocratic/scientific rationale, which is dominated by positivist science as if policies are value-free; a cultural rationale, which refers to the norms and expectations in society and confers messages about the capacity of society to solve collective problems; a social rationale, referring to the social constructions and the categorization of the specific policy target groups; and a political rationale, which relates to the distribution and management of power and reveals how problems have emerged, been contested and been transformed. These competing rationalities are commonly presented by their advocates as “truths” that have a temporal character and are under negotiation.

In ambiguous and complex situations, actors create stories of social reality through a complementary process of naming and framing, where different features from a particular context are selected for attention (Lehoux et al., 2010; Lin, 2003; Nedlund and Nordh, 2018). According to Schön and Rein, the naming and framing carries out the essential problem-setting function by identifying what is wrong and setting the direction for its future transformation, a process wherein the stories of the policy workers make a “normative leap” from “what is” to “what ought to be.” Schön and Rein’s notion of frames is relevant in order to understand the shaping of policy, its policy problem, and its variety of problem frames.

We chose to study problem frames since they may indicate differences in policy positions that could have vital implications in a particular social context and for the shaping of policy. Actors will not usually question dispositions in the treatment of practical problems that are within the practical actions of what they do and how they relate to each other (Nedlund and Garpenby, 2014); rather, actors prefer solutions that are matched to already preferred understandings of a problem that are in line with their own practices and strategic goals. Moreover, as explained by Rein and Schön (1996: 156), the framing of a policy issue always takes place in a specific institutional context that may have its own characteristics and ways of framing issues, and may offer particular roles, channels and norms for discussion and debate. In such a context, the institutional framing of a problem and its immanent solution are often developed into what can, in our view, be named policy styles. Policy styles can be regarded as the modes of operations and the particular approaches, logics or processes that are institutionalized and are related to the way problems are framed. Also, different policy styles are present at the same time since similar problematic situations can be understood and handled in different ways in the same setting (Nedlund and Garpenby, 2014).

## Method

A qualitative study was designed. During the period May to December 2015, a single episode interview study was conducted in one regional health authority (county council) in Sweden. Twenty semi-structured open-ended interviews were conducted by both authors (one author present for each interview): healthcare politicians (5), senior administrators (5), service strategists (5), and unit managers (5). We decided to select actors with different positions and responsibilities with regard to the making of policy—defined by the term of their formal appointment. The politicians were all members of the Central Healthcare Committee (in Swedish: *Hälso- och sjukvårdsnämnden*) and were thus responsible for commissioning health services in primary and hospital care in their regional authority. The sampling of the politicians followed a stratified and convenience strategy where five ordinary members of the committee were approached, as they represented different political parties and were drawn from both the governing majority and the opposition. The sampling of the senior administrators was strategic and stratified. They were all members of the regional authority’s Central Management Group (in Swedish: *Ledningsgruppen*), with roles either as advisors to the politicians or as managers in the health service, some of them with a clinical background. The sampling of the unit managers was stratified as they represented a spectrum of clinical units within the health authority. In the case of the service strategists, the sampling was a mix of stratified and convenience strategy, drawn from different clinical units, and with various backgrounds such as senior nurses or economists.

A topic guide was designed with key themes, such as the present situation in Sweden with regard to the status of patients in current healthcare, patients’ ability to influence their care today, which factors affect the status of patients, the importance of policies and guidelines in this respect, what the situation looks like in one’s own organization, possible conflicts that could appear, handling various situations when encountering patients, and how resource shortages and limit setting affect the status of patients. The interviews followed the “expert interviews” model (Bogner et al., 2009) and the topic guide had an intended flexibility, which implied that it was open to alteration depending on what the interviewees found interesting or when new topics arose (cf. Alvesson, 2011; Bryman, 2012). Follow-up questions were used as a tool to get the conversation started or to explain questions that were not understood by the informants. Informed consent to record the interviews was obtained before each interview. The participants were also informed about how the collected data would be analyzed and presented, with a particular emphasis on the fact that identification of individual informants would not be possible in the final presentation. Each interview lasted 50 to 90 minutes and was carried out at a location chosen by the informant. They were audiotaped and transcribed verbatim. The transcripts were translated from Swedish to English by both authors.

A thematic content analysis of the interviews was carried out in accordance with “The framework approach” (see Ritchie et al., 2003). The framework approach allows analyses in an abductive way (Alvesson, 2011), meaning that there was an interaction for sensitivity to the material, but also allowing the use of pre-existing theories and knowledge. The data analysis began by asking the question “What?” to try to ascertain what was going on and to familiarize ourselves with the data. Subsequently, recurring themes were identified by close paraphrasing (condensation), and a conceptual framework was then created, which covered both the recurring themes and the issues that were raised in the topic guide, sorted under each category of informants. The aim was to authenticate the expressions that originated from the informants. Throughout the process of data collection, analysis patterns and recurrent topics, themes and sub-themes were identified. Even if coding is an important part of the analysis, the interviews should not be seen as a building block for knowledge production, and the ambition of finding specific categories should not stand in the way of creative and critical thinking (Alvesson, 2011). It was an iterative process where the analysis started with the first interview, and where reflections could be brought up in coming interviews following the flexibility of the interview model. The transcribed texts were read several times to reflect and ensure that they corresponded to the analysis.

Since the research focus was on the informants’ capacities as public officials (elected and unelected) and thus in compliance with Swedish legislation and The Act concerning the Ethical Review of Research Involving Humans (SFS, 2003: 460), ethical approval was not needed. The study was conducted in accordance with the guiding ethical principles for research in humanities and social science set out by the Swedish Research Council. All informants were assured of anonymity and confidentiality, and were asked to give their informed consent before answering the questionnaire and being interviewed. Only the research team had access to the raw data, which was kept confidential.

## Findings

In the following section, we highlight how four actor groups as policy-makers perceive the patient as a constructed policy problem in a public healthcare system. The problem frames held by the politicians, the senior administrators, the service strategists and finally the unit managers will be reported on separately (see Table 1). Each account starts with a section on how the actor group contributes to the making of policy in the health authority and ends with a short summary of findings.

**Table 1.** The problem frames held by the informants (actor groups) on the changing patient role, the preferred policy style and labels illustrating a tendency of “scaling up or down.”

Actor group	Problematic situation(s)	Preferred policy style
Politicians	Impossible to resist change due to new societal values (society)	Patients should take more responsibility for their own health and their healthcare (*individual*)
	Personal factors among patients have an increasing impact on care (*individual, system*)	Professionals should change their attitudes toward patients (*professional*)
	Patients are more demanding, and in some cases too demanding (individual)	Give professionals enhanced opportunities for meeting with patients (*system*)
	Patients are still disadvantaged (*system*)	
Senior administrators	Health system adversely affected when patients act as consumers (*system*)	Create an alliance between patients and professionals (*system*)
	Personal factors among patients have an increasing impact on care (*individual, system*)	Pay more attention to continuity of care (*system*)
	Patients who can use new opportunities benefit most (*system*)	Well-informed patients are an asset to healthcare (*individual, system*)
	Patients not always capable of judging information on care (*system, society*)	
Unit managers	Structural barriers exist, preventing patients from getting good care and proper attention (system)	Professionals to regain some power from patients (*professional*)
	Personal factors among patients have an increasing impact on care (*individual, system*)	Healthcare should make better use of patients’ experiences and views (*professional*)
Service strategists	Personal factors among patients have an increasing impact on care (*system*)	Professionals have to listen better to patients (*professional*)
	Inability to listen to patients in the health service (*professional*)	Healthcare should make better use of patients’ own resources (*professional, system*)
	Institutional factors affect professionals’ attitudes and actions *(system*)	Alter the existing power structure in healthcare *(professional, system)*

## The patient viewed as a policy problem in healthcare

### The problem frames held by the politicians

In their making of policy, the Swedish regional healthcare politicians are restricted to establishing general guidelines on the provision of healthcare in different clinical areas. They ultimately make formal decisions on the allocation of resources to broad clinical areas in budget terms.

According to the politicians, the patient role has undergone changes with implications not only for healthcare staff but also for those responsible for overall policy-making and the allocation of resources, that is, the regional politicians. As healthcare cannot remain unaffected by changes in public values, the informants regarded the fluctuating role of patients as part of the transformation of society at large.

“I don’t believe that politicians create it [more demanding patients] but I believe that politicians follow and acknowledge it and thus support current trends in society.” (I1)

“And where we as politicians and those working in healthcare are forced. . . to back down, because the demands and expectations of the citizens are so strong. We are unable to resist it.” (I2)

Politicians interpreted what they were experiencing—that patients are becoming more demanding—as an expression of a situation where people regard themselves more and more as consumers and healthcare as goods to be consumed at their own convenience. They mainly referred to meetings with individual patients, patient organizations, and senior clinical managers.

“I want care, and I want care now.’ And it’s my requirement for care that applies, and then I go to the health center and demand that I get an appointment now. And if I don’t get it, I’ll be very annoyed and disappointed.” (I1)

“I’m sick now and I want it [care]’—and then they get up and sit down outside the emergency room. That’s a current phenomenon. And people react in a way that the healthcare organization is not always built to handle.” (I3)

In the interviews, politicians acknowledged a widening gap between patients having the ability to use sources of knowledge independently to influence their care and patients who continue to be dependent on professionals to navigate their way through the health service. While an increasing number of patients prepare themselves by seeking information on the Internet before visiting a doctor, there are still people who cannot benefit from the new opportunities that information technology offers. Likewise, patients who suffer from chronic diseases are often more knowledgeable about their health than their doctors, but they differ in their ability to communicate their health problems to professionals.

“Under the surface, I would say, it’s much more controversial [patient participation], because the patient is expected to have insights, rather deep knowledge, into his or her care, which takes years for professionals to assimilate.” (I4)

“If you have the knowledge, you can demand more. If you have the language you can speak for yourself, and it’s about your language skills, because if you can’t speak Swedish you are even more left out. It may also depend on which socio-economic environment you come from, even if you speak very good Swedish. What your opportunities are to appear convincing and make demands.” (I1)

The politicians regarded prevailing norms among healthcare staff as a problem, as this could make professionals unwilling to respond to new demands from patients. Hence patients were also seen as being disadvantaged in relation to healthcare staff.

“I think, I believe that most people feel left out. When you’re a patient, you are sick. You. . . are in the hands of someone, a system, an organization you become dependent on. . . their ability to take care of. . . It will of course place you in a relationship of dependency.” (I1)

“The doctor has to step down from the pedestal and be on same level as the patient in a completely different way than if there’s a patient who does not know anything.” (I3)

The politicians believed that in the present climate where some patients become stronger—and some weaker—the staff will also suffer most due to pressure in different directions.

“Yes, as I said before, one group [of patients] will be very much stronger, one group becomes weaker and the weakest of them all will be the healthcare staff.” (I4)

“. . . the patient thinks that he or she should control what care he or she should have. Thus, in a peremptory way.” (I1)

When reflecting on how they as policy-makers should strive to create a proper interface between patients and the health service, the politicians displayed a high degree of uncertainty. Some pushed the responsibility to the patients, claiming that they had to be more “realistic” about what they could expect from the health service.

“It depends on what one means by one’s own choices. . . I mean that as a patient today you have. . . it’s always you who choose yourself, because most diseases are linked to how we live, how we take responsibility for our own health and our own illness.” (I2)

“Patients need to become even more involved, take greater responsibility, share responsibility. Have a good dialog between those responsible for the treatment and the patient, so that they interact in the treatment. I think it is very important for recovery and a good result. [. . .] Patients should take greater responsibility for their self-care, I believe, and all of us in society can take greater responsibility.” (I1)

The interviewees also referred to structural measures in the health service in order to influence the conditions for the meeting between patients and healthcare staff.

“As a politician, what you see and think is that it is necessary to do more; we should provide incentives to change, for example, the amount of documentation [in healthcare] to increase the time with the patient.” (I1)

“The personal meeting is very important, and it should be good. Yes and then that. . . that the care system and the staff see the patient as a whole person and not just this particular disease or overall disease history, but they look at the life situation as a whole, and look at the whole person, the different parts that are required to make this as good as possible.” (I5)

As the politicians are laypeople elected to represent the public in a particular geographical area (corresponding to the health authority) they draw on different sources outside and inside the healthcare system. Some have worked in healthcare themselves and have experiences that they refer to. In the interviews they also mention their own experiences as patients or experiences of healthcare among family members, relatives, and friends. As elected representatives, they discuss matters within their party group which they point to as well as input from national statistics on how patients experience healthcare. They also mention meetings with patient organizations (which they commonly refer to as “complaints”) and organized “patient dialogs,” where individual patients suffering from different conditions convey their specific experiences directly to politicians. One additional source that they refer to is meetings with senior clinical managers to discuss the healthcare budget. Occasionally they refer to input from clinical units where meetings with unit managers and staff have been organized on specific topics.

### The problem frames held by the senior administrators

The senior administrators in this study are either top-level strategists in the health authority advising the politicians or are responsible for operational service planning at upper clinical levels. In terms of policy-making, this is accomplished through general strategy discussions or dialog on specific issues with the politicians or the clinical managers in the line organization of the health authority.

According to the senior administrators, those patients who regard themselves as consumers and healthcare as a commodity that can be consumed quickly and at their convenience are the ones who benefit most from the current trend where opportunities to get quick access to care have been strengthened.

“I think, on the other hand, that there’s more of a tendency to look at care as something taken for granted, which one wants to consume differently than one did before, and that, unfortunately, it feels that in some cases one starts to look at healthcare services in the same way as other services.” (I6)

The interviewees referred to a large group of patients who have other needs related to the consumption of care. One aspect that was highlighted was continuity of care, which was seen as a prerequisite for what the informants regarded as desirable: a more person-centerd meeting with the patient.

“To some extent, the patient’s need for continuity has been given a lower priority than the idea of accessibility, where the main thing is to get care quickly. . . but there are two things I believe most patients want: a trustworthy doctor, whom you know you can turn to, and to get care quickly when they need it. . . I think we will have to work harder with continuity, I think it will be a requirement too.” (I7)

“But there is so much to do with the relationship in the patient meeting or in additional meetings, if you have continuity, which I think is very important. And if you have continuity of care, you can also see the individual, what this person’s resources are, what ability the person has to understand what we are talking about.” (I8)

The administrators expressed concern about a situation where considerable differences exist between service areas with regard to how patient participation has advanced as well as how individual patients will be able to benefit from the current changes. They often referred to increasing local political demands as well as national political initiatives.

“All the e-health services that will be made available to the patient, which I think is great, but which are of benefit to those who have the ability to use them. But here we will experience an unfair situation in future, because you’ll have those who will be able to utilize e-health services and then you’ll have a group of people, most the elderly and those with different disabilities, who will not be able to use them.” (I9)

“Of course, the patient’s own ability. . . to interact, if you’re eloquent, have good knowledge, are able to ask questions, dare to stand up to someone who can be perceived as an authority, of course it matters.” (I6)

In general, the administrators viewed the well prepared and knowledgeable patient as an asset to the health service and to society at large. Knowledgeable patients were seen as more able to follow-up their own care and thus able to supervise the quality of their own care.

“If we have patients who are co-producers and we build an alliance with the patients, we will get better results.” (I10)

“Diabetics who check their condition, their treatment, monitor their values, maybe submit reports, have contact, and maybe have a video conference with the health service. Such things will definitely increase in the future and then the patient becomes the one who has. . . is the one who has the control to a much greater extent.” (I7)

“I also think that they [the providers] have understood the importance of a well-informed patient, who is much easier to treat.” (I9)

Still, the senior administrators emphasized the importance of professional knowledge and expressed doubts about the quality of information that patients are able to access by themselves, for example, on the Internet, and thus their ability to use information to make well-informed choices about their own care.

“And then, I think it’s because, you must see that, you cannot just go in and demand something, but you have to work with this too, so you get an understanding of what it is. What assessment you have been doing on the medical grounds of what the patient should have.” (I 10)

“Unfortunately, it seems that in some cases you [the patients] begin to compare health services with other services, which you may perceive to some extent as having the same effect. I mean, the interest in alternative medicine, and such things. I think you begin to experience, rightly or wrongly, a somewhat more relativistic view of the services and the knowledge that the healthcare system offers.” (I6)

A key to future healthcare, according to the administrators, is the formation of a workable alliance between patients and professionals. This was seen, however, to be a tricky balance: listening to patients better, but having to remain professional.

“And to be more personally responsible as a patient, it must be connected to me as a provider of care too. . . partly to make it clearer, it’s an increased joint responsibility as well as moving from being a healthcare provider to more of a consultant or a support function that delivers certain parts [of your care], I think it’s a change, a cultural change.” (I6)

“I think this will definitely increase and then the patient becomes the one who has more. . . is the one who has the control to a much greater extent. Nevertheless, it’s a fact that the health service must work for everyone. . . there’s a need for different capacities for different reasons. It’s important that healthcare is responsive to how the individual patient wants it and needs it.” (I7)

Obviously, some of the senior administrators had backgrounds as health professionals with their own experiences of how patients interact with healthcare, which they mention as a source of knowledge. Apart from this, they frequently mentioned their meetings with managers at the clinical units and politicians for knowledge exchanges at regional level, which they often refer to. They made several remarks about how the politicians used to convey ideas and attitudes in very general terms about a preferable course of action within the health authority, which they have to interpret, relate to and implement, although sometimes they had doubts about what was best for the health service and its patients.

## The problem frames held by the service strategists

The making of policy by service strategists has a multi-level character, as they (a) are in close contact with the clinical units and (b) interact with the administrative branch of the health authority. Service strategists in the health authority primarily contribute to policy-making by conveying evidence (originating from both research and the practice level) at meetings with clinical units (unit managers and healthcare staff) and the administrative level (senior administrators and politicians).

The service strategists expressed concern about differences in capability among patients in combination with the current drive toward extended patient participation. According to the informants, there is a long way to go before the health service knows how to tackle the disparities in resources and abilities among patients.

“I believe that the demands we place on accessibility don’t benefit patients who have chronic diseases, because they sometimes have to take a lower priority than the urgent cases, in primary care, seven days and so on [referring to the Swedish waiting time guarantee]. So I think that requirements for accessibility have somehow made it better for some groups, but worse for other groups.” (I11)

“Those who are weak, who feel weak in relation to the system when they enter the system or do not have the prerequisites and have various communicative difficulties or impaired autonomy or multi-sick patients, and if they do not have a representative, then you do not have the same opportunities. But then again, I’ve touched upon it earlier, the health service has not created any opportunities for patients to take their place either.” (I12)

The service strategists emphasized that health professionals must learn to listen more attentively to patients in order to benefit from their experiences and their knowledge. Patients in general are knowledgeable, and are able to reflect and express their views if professionals are willing to listen. An inability to listen will make the health service less effective and will thus result in unnecessary interventions and readmissions.

“And a patient today can probably be much more of an expert on his own illness than the patient could historically. This is quite new; in the past, the doctors had the expert role, to a much greater extent, and the doctor’s role still is the expert role, absolutely. But the patient has his own possibilities, which was not the case before.” (I13)

“So you could reach even further, to get more personalized care, where the patient may not think that he or she needs all the interventions that a healthcare programme advocates, because of a different life situation.” (I14)

It was foremost the service strategists who highlighted institutional factors, which according to them have an impact on sustaining prevailing norms in healthcare, and make it difficult for professionals to listen to patients. In this respect they drew on both their own close contacts with healthcare staff and national studies (frequently referred to as “evidence” and seen as important when interacting with doctors and nurses).

“It’s the nurses who come into contact with most patients during their care, and what works against the nurse is the hierarchy within the hospital. And it can be very, very hard for a nurse to bring up hygiene, to remind those higher up in the hierarchy about the patient’s experience unless they share the same attitude. It can be a very difficult role to have as a nurse. And it may be very difficult to question superiors in the hospital hierarchy.” (I13)

“But when I meet—when I work with healthcare professionals, I feel that they are very. . . they are very, as it were, fixed on what they do, and sometimes I feel that it’s hard for them to imagine the patient’s situation; what the patient is actually experiencing. They are very much here and now, and ‘this is how we work,’ and then they take it for granted that if they improve things they do, then it will be better for the patient, and it’s not actually the case.” (I15)

According to the service strategists, it ultimately ends up with the question of who should be in control of what in healthcare, the professionals or the patients, and who has the power to decide.

“But that’s what I experience when I discuss this with healthcare professionals: ‘Yes, but patients cannot always have it the way they want,’ and very much in our system, it is in some way about opposing the patients, keeping them away. This sounds terrible, but so many people want to get access so someone has to make the cruel priority.” (I15)

“I don’t want to sound pessimistic, but this is about who has, you know, who has the power to formulate the question, it’s about resources and the power to change. And that power has not yet shifted to the patient.” (I12)

The service strategists mentioned information about topics which they found relevant, for example, personalized care, originating from documents published by research institutes, universities, and other knowledge generating bodies. Most of their impressions, which they referred to, however, had their origins in direct contact with local clinical units, their patients and staff. Situations and experiences raised in the interviews were mostly based on their own observations of what is going on at “the street-level of care,” when talking to practitioners.

## The problem frames held by the unit managers

As providers of clinical care, the unit managers are responsible for transforming contracts and directives from commissioners into service plans using the monetary and human resources at their disposal. Their making of policy is carried out through budget setting, service planning, and dialog with and instructions to healthcare staff. They can affect overall policy in the health authority indirectly by putting forward practice-based and scientific evidence through the line organizations to reach politicians and commissioners.

The unit managers highlighted the growing discrepancy between what the health service is supposed to achieve and the actual conditions for delivering services to patients. In this respect, they were referring to what came up in the ongoing dialog with their staff. One example is the right for patients to be listened to, which has been further enhanced by new legislation, but where healthcare staff were said to be experiencing difficulties in bringing about an improved situation. The unit managers gave various reasons why an ideal situation was hard to accomplish.

“But at the same time, reality seems to work against it, if there are doctors who are more dispersed, do not work full time, have other assignments and so on. Then it will be harder to live up to. . . what are often good, sensible suggestions about how we should work and how we should behave. So there’s a discrepancy between what we expect to do and what resources we have, as it were, in everyday life to live up to it.” (I16)

“Then there’s a very, very, very high volume of administration, and this takes up a lot of competent staff’s time, and very many external assignments we’ll have to do, so we feel, I feel. . . and all of us working here feel that the time with the patient is becoming more limited. So, I feel that we should focus more on the patient, and get more help with related activities than we have today, and this would make it easier to use the skills correctly, I think.” (I17)

When the situation is changing, from one where professionals are in total command to one where patients are encouraged to take an active role, tendencies that previously were not fully recognizable will become visible. Accordingly, the unit managers explained that there is a wide variation between service areas and individuals with regard to what patients are able to gain from the health service. Most of all, differences in resources between individuals will have greater consequences, as patients are more often encouraged to speak for themselves and make their own choices independently of professionals.

“In the past, it was probably possible for the patient to participate, at least in the service area where I used to work, you allowed the patient to participate, but they had to take the initiative. Now it’s more that we take the initiative to make them [the patients] participate.” (I18)

“It’s also the case that in the present system it’s easy to get help and support with simple measures, with problems that can be easily solved, while I feel that patients with more chronic conditions and who are able to bring their own case forward, or who may not have strong spokespersons, for them it’s harder to make progress in the system.” (I19)

Some unit managers explained that they had witnessed a shift in the balance of power between professionals and patients that they saw as barely positive. As more patients have distinct views on what they expect from the health service, there is a risk that they will be disappointed if the services they expect cannot be delivered. Accordingly, there are professionals who feel that the shift in power should be matched with changes in patient responsibilities and that professionals even have to regain power.

“So I think it’s important that we dare to take back some power again and that we dare to resist demands and dare to stand up for the judgments we make. We are a little afraid of this today, I believe.” (I17)

“If a patient is to be involved in choosing between one treatment and another, but one treatment does not work, is it our responsibility to say that it’s OK to use that treatment? Or should we take full responsibility by saying that then you must take the other treatment, say tablets or injections, or can we say that it’s your [the patient’s] own responsibility to handle the treatment?” (I18)

Some interviewees acknowledged a need for the health service to improve the meeting with patients by obtaining information more systematically about what they expect, not only about the content of care, but also about the way care should be organized and delivered.

“Yes, we should have more patients present in the evaluations; we would need to have patients who could assess whether this works. Does it work well or does it not work well? We would simply like to have such measurements, someone who sits down to call [patients] continuously to find out how well it actually works.” (I19)

“I’d like to have a continuous dialog, a continuous evaluation, ongoing advice that one could have in a dialog so that one could improve over time, all the time.” (I20)

The unit managers referred to meetings with representatives from clinical units in other parts of the country to exchange information and experiences. They also mentioned the national patient survey, which reports on how patients experience encounters with healthcare. The unit managers described the interactions with patients in their own clinical units, which they said gave them lots of insights, as being more important. The interviews, however, illustrated how the ways in which the clinical units interact with patients vary, from short meetings to life-long contact. One important source, which they frequently referred to, was interactions with the staff that gave them information about day-to-day work but also carry forward ideas of how clinical tasks can best be accomplished.

## Discussion

In recent decades, the perception of the appropriate interface between the patient and the healthcare system has changed. This is happening in many countries, where both patients and providers have started to regard healthcare differently than was the case just a few decades ago. In National Health Service-type systems, like the Swedish one, that have traditionally been provider-oriented and organized to deliver standardized care on equal terms for all, the patient as a policy problem will become more articulated. This is because healthcare is very much at the center of politics, and shifts—expected or unexpected—that affect the interface between patients and the system will have implications for policy-makers, whether elected or unelected.

**Figure 1.**
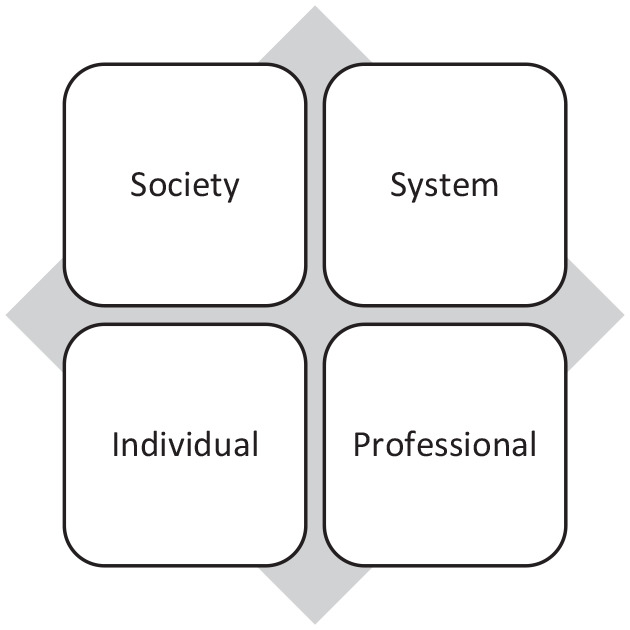
Different accounts that appear in the framing by the actor groups.

Our case illustrates how the various actors approach the constructed problem differently, having different problem frames. Drawing on Nedlund and Garpenby (2014), we can see that the actors relate not only to different problem frames but also to the inherently different *policy styles*. This becomes even more visible when the framing is disentangled with regard to which perspective they employ in relation to different accounts: society or the individual, or the (healthcare) system or the (healthcare) professional (Figure 1). Accordingly, the labels used in Table 1 are intended to illustrate how the framing by the actors represents the different accounts and thus also the shift between these, such as what could be seen as a scaling up or down of a problematic situation.

Looking at the politicians, what we see in their structuring of problems is how they dwell on all societal perspectives. This can be seen as an indication of how their problem-framing is taking place in an institutional context that differs from that of the other actor groups. Politicians in their role are inclined to move more freely between the different perspectives (society, individual, system, professional). They use a systemic perspective, but in contrast to other actor groups, they also focus more obvious on the individual (patient/citizen) and on society. As policy-makers, their impression of healthcare and its patients are influenced by many different sources.

Problem frames among the politicians relate to Crouch’s (2000) notion of “post-democracy,” that is, politicians are inclined to give in to what their “customers” want, adhering to a consumerist trend in Sweden. As greater consumer choice is virtually irrefutable in Swedish healthcare today, the politicians prefer to highlight the importance of patients as individuals taking more responsibility for their own actions. There are also, however, some politicians who are more inclined to solve possible misfits in the present system by focusing on the professionals—they should change their attitude toward patients.

It is the senior administrators who see the consequences most clearly when publicly funded healthcare adapts to a situation where patients are increasingly allowed to act as consumers (Toth, 2010). While endorsing increased opportunities for patients to participate in their own care, they question a health service where patients foremost act as consumers. Although the administrators refer to both patients and professionals, as policy-makers they generally apply a systemic perspective in their problem-framing and in the policy style they represent.

The service strategists, who have insights from both high level policy-making and clinical practice, frame problems merely as being related to inconsistencies between the different actors in the healthcare system. They have a strong systemic perspective that draws on insights from the professional world. Hence, they highlight institutional factors and the strong norm system in healthcare, and also choose to frame problems in terms of the power structure.

The unit managers, who are responsible for care provision at the micro level, also have identify conflicts, but of a different magnitude: between performance-related economic incentives and other demands (such as getting involved in extensive documentation of care) which, in their opinion, make it difficult to enhance the dialog with patients. Their way of framing problems is mainly in terms of systemic barriers. This is an actor group with a strong systemic perspective, constantly affected by the need to respond to requests from politicians, administrators, patients, and their staff. On the whole, the unit managers prefer to solve problems at the micro level, where professionals are in control. However, there are examples where they express an interest in communicating with patients as a group in a manner that is more systematic, but is interpreted as useful mainly from a professional perspective.

It is in the preferred policy style that the differences between the actor groups emerge most clearly when the framing follows systemic and professional accounts.

Why is that? Part of the explanation is in the meeting between a highly dualistic healthcare system and various rationales that the actor groups consider to be the “truth” and the “answer” to problematic situations. Also, the organization and the framings of policy problems feature various competing rationales—technocratic/scientific, cultural, social, and political—encapsulated in policies (following the taxonomy of Nedlund and Nordh, 2018) that become social realities for the advocated actors. What might appear confusing is when actor groups seem to name and frame problems in a similar fashion, despite the fact that they clearly have different roles and policy positions. Their understanding of a policy problem is to certain extent influenced by their belonging to the same institutional context (the regional health authority). Following Rein and Schön (1996), this context has its own characteristics, channels and norms for discussion and thus makes its imprints on framing activities. Apart from this, there are real differences in their background, experiences, and policy positions that also affect their perspectives and how they connect to the various rationales.

The framing of the patient as a policy problem is due to the nature of the Swedish healthcare system, being both an action organization, deriving its legitimacy mainly from the concrete output (applying science and evidence), and a political organization, deriving legitimacy chiefly from formal structures and decisions (as part of a democratic society) (Brunsson, 2002). As Harrison and McDonald (2009) note, elected policy-makers in healthcare are torn between satisfying voters and avoiding unmanageable conflicts in the organization they are elected to govern. Our politicians, who are ultimately responsible for healthcare as an action organization, understand that they have to strike a balance between defending overall principles guiding Swedish healthcare, that is, delivery of care according to need and evidence, and the opinions and requirements of professionals and patients, following a technocratic/scientific and a cultural rationale. The balancing act between different requirements is a hallmark of the modern healthcare system (Russell and Greenhalgh, 2014). However, the politicians are not afraid to blame both citizens/patients and professionals—patients can be too demanding and should take more responsibility, and professionals should change their attitude. Framing in the form of blame shifting can be seen when politicians refer to societal values as being impossible to resist; it is implicitly not our fault, there is nothing we can do about it. Using a “blame game” (Garpenby and Nedlund, 2016; Hood, 2011) is an illustration of how a political rationale is present in the framing of problems, but the politicians’ problem-framing also falls within other rationales. When referring to patients as being too demanding, this is an example of how politicians end up in a culture rationale—the norm to date has been a humbler patient. Likewise, when patients are referred to as being disadvantaged, this could be seen as an example of a social rationale—how and when somebody is disadvantaged can easily be constructed and reconstructed.

One obvious solution might be to strengthen both patients and professionals. As they are keen not to disturb the order of the action organization, politicians are less inclined to intervene to read just what could be regarded as imbalances in power. However, their understanding of policy problems follows on from how they interpret their reality and the means at their disposal. It is also a question of how they understand their strategic goals. Ultimately, this is a question of what their source of legitimation is: the action organization or the political organization, or both. This could be a reason why politicians shift accounts and why there is a distinguishable scaling up and down in their problem-framing.

Among the senior administrators, problem-framing focuses on efforts to prevent patients from being misinformed, and thus the preferred policy style will be to have patients with the right kind of information, as this will make them “better” patients for co-production in the action organization. It is a cultural (in their case an organizational) rationale that shines through in their framing—which norms and expectations there are among those in a position to administer this system (the action organization), who have to balance its many goals and principles (the political organizations) and work out practical solutions. In this, we can see a streak of a political rationale when the administrators point to a health system that is adversely affected by those who are ultimately in power: the politicians who have opened up to consumerist behavior, disturbing the existing order.

The unit managers prefer a policy style where patient participation as co-production is accepted up to a certain limit, as they also indicate that the balance of power between patients and professionals needs to be stabilized, and may already have shifted too far in favour of the former. Clearly they manage healthcare as an action organization but are policy-makers within a political organization. In this environment of competing interests, their problem-framing is articulated in terms of a political rationale. In their world, patients are denied proper attention due to forces that are powerful and thus beyond the control of both patients and professionals. What appears in the preferred policy style is also a matter of power—to alter this or allow patients voices to be listened to.

Interestingly, the service strategists, who are involved in the implementation of new policy initiatives, seem to favour a policy style aimed at the professionals; their attitude toward patients and their willingness to let patients participate, even if this could disrupt the existing power structure, as in their view this is the key to participative initiatives. In this we can see social and political rationales, such as in the categorization of the specific policy target groups as well as the distribution and management of power. A cultural rationale is also displayed, as they refer in their problem-framing to both inherent factors in the healthcare system and attitudes among healthcare workers.

In this study on how four actor groups frame problems related to the interface between healthcare and its patients, there are similarities in their accounts of what constitutes problems. Exactly how startling is this finding? The traditional way of looking at various categories of policy-makers in healthcare, for example, politicians, administrators, managers, and frontline health workers, is to emphasize how different they are in their roles—as if they were isolated in different worlds (Svara, 2006). Clearly, our actor groups have different roles, experiences, and grounds for legitimacy (electoral mandate, legal and political astuteness, scientific, and other knowledge, etc.). This context (the regional health authority) is characterized by its intricate power dynamic, where on the one hand there are actor specific interests and norms, and on the other actor interdependence in preserving the balance between the political and the action organization.

Furthermore, the actors belong to the same policy arena (the regional health authority), that is, they are part of the same institutional context, where similar policy sources are at play: documents, statements, meetings, local media, etc. As part of the same institutional context they share similar but not totally overlapping “mental models,” that is, what Klein and Marmor (2008), drawing on Vickers (1965), name “assumptive worlds” for what is a problem, what causes it and what solutions there might be. In that way, the similarities and differences in this study could be seen as reasonable.

Problem-framing is inherent in the making of policy everywhere, irrespective of country and healthcare system, and is thus general in its nature. However, depending on which institutional context we are looking at, there might be some specific national traits. In the decentralized and regionalized Swedish system, various kinds of policy-makers—whether elected or unelected—are linked more closely than may be the case in a public system like the British NHS, where the policy actors are structured in other ways. This could have implications for what our interviews show and which frames will develop. Problem-framing among policy-makers could also be influenced by the current national debate on topics such as patient rights, patient power and the allocation of resources to various branches of healthcare, but also by which solutions and policy styles have been used in the past.
